# Sedation and analgesia in the trauma intensive care unit of Inkosi Albert Luthuli Central Hospital – the effect of anti-retroviral therapy: A retrospective chart analysis

**DOI:** 10.1007/s00068-024-02639-z

**Published:** 2024-08-28

**Authors:** O. G. Mngoma, T. C. Hardcastle, K. De Vasconcellos

**Affiliations:** 1 University of KwaZulu-Natal, Durban, South Africa; 2 Inkosi Albert Luthuli Central Hospital, Durban, South Africa; 3 King Edward 8th Hospital ICU, Durban, South Africa

**Keywords:** Trauma ICU, Sedation, Antiretrovirals

## Abstract

**Purpose:**

Adequate access to antiretrovirals (ARV) has improved the longevity and quality of life of people living with the human immunodeficiency virus(HIV). Antiretrovirals are known to cause multiple drug–drug interactions. It was noted clinically that patients on ARVs appeared to be more difficult to sedate. This begs the question of the clinical impact of these drug interactions, should clinicians adjust sedative dosages when managing patients on ARVs? This study aimed to investigate the presence of and measure the differences in sedation and analgesic utilisation between polytrauma patients on ARVs and those not on ARVs.

**Methods:**

This retrospective observational chart review included consecutive adult polytrauma patients admitted to the Trauma ICU IALCH between January 2016 and December 2019. HIV status and ARV use was documented. The total sedation per drug utilised at 24, 48 and 72-hour interval was calculated and tabulated accordingly. Drug utilisation was compared to ARV status.

**Results:**

A total of 216 adult polytrauma patients were included in the study. A total of 44 patients were HIV positive and 172 were HIV negative. Of the HIV positive patients 41 (93.2%) were on ARVs. Multiple comparisons were confirmed, however the primary analysis compared HIV negative patients with HIV positive patients on ARV. Total morphine, ketamine, midazolam and propofol doses were all numerically greater in patients on ARVs, although none of these reached statistical significance. The use of morphine rescue boluses during the first 72 h of ICU admission and the doses of ketamine and propofol on ICU day 3 were significantly greater in those on ARVs.

**Conclusion:**

The data analysis showed that patients on ARVs required higher doses of some analgesia and sedation in ICU and lower doses of midazolam. This needs to be considered when sedating patients in a setting with a high HIV prevalence.

## Introduction

Polytrauma and HIV are significant challenges for the healthcare sector in lower- and middle-income countries (LMIC). The primary causes of trauma are interpersonal violence and inadequate transportation infrastructure [1]. It is a well-established fact that critically ill polytrauma patients endure significant pain, ranging from mild to severe, throughout their stay in the intensive care unit (ICU). This pain arises from both their injuries and the necessary medical interventions performed on them. The management of agitation in the ICU environment is further complicated by the need to limit light and noise [2]. The Richmond Agitation Sedation Score and Ramsay Sedation Score are widely utilized and scientifically verified instruments for evaluating and tracking the degree of sedation in the intensive care unit (ICU). The ICU setting is widely regarded as very stimulating due to the presence of intubation, general care procedures, and incessant alarms that cannot be muted [3–5]. The frequently utilized medications in the ICU include opioids such as morphine and fentanyl, hypnotics including ketamine, propofol, and thiopentone, as well as midazolam, dexmedetomidine, and clonidine. Haloperidol is utilized as a second or third choice tranquilizer [5].

The management of trauma, along with the presence of comorbid diseases such as HIV/TB and psychiatric disorders in the intensive care unit (ICU), results in the use of multiple medications (polypharmacy) and an increased likelihood of significant drug interactions. Literature has documented the adverse consequences linked to insufficient and excessively aggressive pain and agitation control. Medical practitioners in trauma units must be aware of the potential changes in the way medications are processed in the body, known as altered pharmacokinetics, because of the patients’ medication history [6].

### Drug interactions between sedatives and ARVs

Antiretroviral drugs (ARVs) have the potential to affect the metabolism and therapeutic levels of other medications, making them a chronic medication of importance to the intensivist and trauma surgeon due to the link between risk-taking behaviour, trauma and HIV/AIDS. Among the different classes of ARVs, NNRTIs and PIs especially Ritonavir are known to affect metabolism of other drugs by inhibiting or stimulating the Cytochrome p450 enzyme activity [7]. The commonest enzyme pathway responsible the drug interactions and expected clinical outcomes is the 3A4 as indicated in Table 1 below, it is vital to note that no drug interactions are expected between NRTIs and ARVs. No study conducted in South Africa has particularly examined the therapeutic impact of providing sedatives to patients who are on antiretroviral medications. In 2020 a national study was conducted by the university of Cape town investigating health care workers knowledge of drug interactions associated with dolutegravir. This study showed that there are major gaps in knowledge of drug interactions associated ARVs amongst primary health care workers [8]. An important learning point in this study is that coadministration of dolutegravir can cause significant adverse reactions. This can be catastrophic in the acute setting as well as on a larger scale when considering the altered pharmacokinetics and the risk of causing ARV drug resistance.

**Table 1:** Indicates the expected pharmacokinetics between commonly used sedatives and ARVs. [7, 9–12]

Commonly used sedatives	CYP 450 enzyme induction/inhibition or substrate				Expected clinical outcome
	NRTIs	NNRTIs (3A4, 2B6, 2C9, 2C19,2A6	PIs (3A4, 2D6,2C19, 1A2, 2C9	INSTIs (3A4, 2C9, UGT1A1)	
**Morphine**	-	CYP 3A4	2D6,3A4	3A4	Efavirenz reduces its metabolism, risk of toxicity of active and inactive metabolites
**Ketamine**	-	3A4	3A4	3A4	
**Midazolam**	-	3A4	3A4	-	Potentiated sedation if co-administered with a PI, cobicistat and Elvitegravir due CYP enzyme inhibition. Therefore a lower dose of midazolam is recommended. Decreased midazolam concentration if co-administered with Etravirine due to enzyme induction thus a midazolam dose increase may be necessary.
**Diazepam**	-	3A4, 2C19	3A4 inhibition 2C19		PI will cause excessive sedation EFV decreases diazepam concentrations
**Haloperidol**	-	3A4	3A4,2D6,1A2	3A4	PI boosters can moderately increase the haloperidol concentration and consequently its side effects.
**Propofol**	-	2B6,2C19	1A2, 2C19	UGT1A1,1A9	Metabolised by 2B6, 2E1,

NNRTIS = Non-nucleoside reverse transcriptase inhibitors: examples are Efavirenz ( EFV), Nevirapine (NVP), Delavirdine (DLV). NRTI = Nucleoside reverse transcriptase inhibitors examples are Lamivudine (3TC), Stavudine (D4T), Abacavir (ABC), Didanosine (DDI), Zidovudine (AZT), Emtricitabine (ECT), Tenofovir (TDF). PI = Protease inhibitors e.g., Lopinavir/ ritonavir (LPV/RTV) also known as Kaletra, Ritonavir (Norvir), Saquinavir (Invirase), Nelfiravir (Viracept). INSTIs = Integrase strand transfer inhibitors. Dolutegravir, Elvitegravir and Raltegravir

## Methodology

This was a retrospective observational chart-review study, investigating the difference of sedation requirements in patients on ARVs versus those not on ARVs at the IALCH Trauma Society of SA Level 1 Accredited Trauma unit trauma ICU between 2016 and 2019. The trauma ICU is a closed unit in the only quaternary academic hospital based in KwaZulu-Natal. It is an 8–10 bedded unit specifically for trauma patients that services approximately 11.5 million population, admitting both adults and children. The monthly turnover is approximately 24 patients per month. The nursing capacity also influences the admission capacity as a result more often than not, it functions at 8 bed capacity.

As a referral centre most patients were transferred from other institutions or other units in the hospital, much fewer came straight from scene. Analgosedation drugs are administered as continuous infusions titrated according to protocol, with clinical adjustments as needed.

Data was collected from the trauma registry and the electronic patient records. All patients in the trauma registry were screened for inclusion in the study. Patients were excluded if they were under 13 years old, their HIV status was not known, had an ICU length of stay (LOS) of < 72 h or did not receive analgosedation during the first 72 h of their ICU stay. All data collected was entered onto an Excel spreadsheet. Input data included demographic data, data on HIV status and ARV, and clinical data. Outcome data included the use of sedative and analgesic agents and the doses of these agents. Doses were recorded as the total amount of each drug administered in each of the first three 24-hour periods of the first 72 h of ICU admission (ICU-D1 = first 24 h, ICU-D2 = 25–48 h, and ICU-D3 = 48–72 h of the ICU stay). Additional rescue boluses administered in the case of drug infusions were recorded separately. The total drug usage for the first 72 h was also calculated. To avoid errors and omission of information doctor’s notes were cross checked with nursing records as a means of veracity and validity. Drugs considered to be analgosedatives included all potent opioids, all benzodiazepines, ketamine, propofol, antipsychotics, antidepressants and alpha-2 agonists. Simple analgesics e.g. paracetamol and NSAIDS were not included. Due to difficulties in comparing intubated to non-intubated Glasgow Coma Scale (GCS) scores, a decision was made to present GCS as a percentage of the maximum possible score,15 for non-intubated scores and 10 for intubated scores. The patients who came in with known liver disease were documented as this impacts their drug metabolism, liver disease noted were mainly alcoholic liver disease. The ICU beds are equipped with the weight measuring function which allowed documentation and usage of patients weight in calculations necessary. The Ramsay sedation score was used in the unit by both nurses and physician to optimise sedation according to individual needs of each patient as decided upon during the twice daily consultant- rounds done in the unit. The targeted score was 5 or 6 as most patients were either requiring neuroprotection or the in the perioperative phase of acute polytrauma care.

Statistical planning determined a minimum sample size of 196 participants with known HIV status. Data was collected for an additional 20 patients (to allow for any subsequent exclusions) and thus 216 patients were initially included. Statistical analysis was performed using IBM SPSS for Windows, Version 29.0. [IBM, Armonk, NY] Categorical variables were described as numbers and percentages and compared using the Chi-square test or Fisher’s exact test, as appropriate. Continuous data were described using median and interquartile range (IQR) and compared using the Mann-Whitney U-test. A p-value of < 0.05 was considered as significant.

### Inclusion criteria

1. All polytrauma above the age of 13.
2. Patient must remained sedated for the minimum of 48 h of admission.
3. Patient’s HIV status must be known before or during admission period.
4. Minimum sample size reached or exceeded.

### Exclusion criteria

1. Patients under 13 years of age.
2. Medical patients, non-trauma admission.
3. HIV status unknown and remained so until discharge.
4. Admissions that were not for sedated over the first 48 h.
5. HIV positive patients not on ARVs.

#### Ethical considerations

Ethical permission for the study was obtained from the University of Kwa-Zulu Natal Biomedical Research Ethics Committee as a sub-study of BCA207/09 and a waiver of informed consent was given due to the retrospective nature of the study.

## Results

Chart review identified 216 participants with known HIV status during the study period out of a total 1087 TICU admissions (19.9%), with overall 405 excluded due to age < 13, no sedation given, non-trauma pathology, not on ARVs, due to unknown HIV-status and ICU stay less than 24 h. The other 468 charts were not reviewed due to time constraints as the minimum sample size had been reached. The majority of patients sustained multiple injuries and thus required multiple emergency operations and consequently a higher usage of blood and blood products during resuscitation and intraoperatively. The ISS and NISS scores were documented as follows: The whole sample size had a median ISS score of 29 with IQR = 20–38 and the NISS median was 30 with IQR = 22–41. Comparing the trauma scores between the patients on ARVs and those not on ARVs noted median ISS was the same and equal to 29; NISS median 32 and 26, with IQR 25–38 and 22–43 respectively. The percentage of patients who had emergency surgeries was 97.7%, some had one procedure while others had more than one, hence the percentages exceed 100%. Of these emergency surgeries performed 42.2% were laparotomies, with craniotomies (44.5%) orthopaedics (50.7%) and fewer (16.6%) cases of vascular, soft tissue and urology. Blood transfusions were administered to 55.1% of the whole cohort.

Baseline demographic and disease data is presented in Table 2. Due to the small number of HIV positive patients not on ARVs (3 patients or 1.4% of the cohort) we decided to exclude these patients from further analysis. The rationale was that they potentially represented a unique subset of patients and thus could not be analysed with either the HIV negative patients or the patients on ARVs but there were not enough patients to allow for statistically valid comparisons as a standalone subgroup. All comparative data presented is thus for the 213 patients who were either HIV negative or HIV positive and on ARVs.

The most common sedatives administered were morphine, ketamine and midazolam in 96.3%, 81.9% and 23.1% of the cohort respectively. Other sedatives administered were propofol in 9.3% and haloperidol in 4.6% of the whole sample. Comparing the HIV negative group vs. the HIV positive group on ARVs: morphine doses were the same in the first 24 h, however in the 24–48 h interval there was a higher dose requirement by patients on ARVs (*p* = 0.007). The doses required in the 48-72-hour interval, however, were the same. Morphine bolus doses used by patients were also significantly higher in patients on ARVs (*p* = 0.002). The median morphine dose for the whole cohort was 2.3 mg/kg, and patients on ART required a dose 0.7 mg/kg higher than those who were HIV negative, although this was not statistically significant. The cohort ketamine median dose was 31.6 mg/kg and each 24-hour total dose was numerically higher in patients on ARVs. Statistical significance was noted though on D2 and D3 (*p* = 0.043 and *p* = 0.004) respectively. A lower proportion of HIV negative patients required benzodiazepines, although the median dose of midazolam administered was higher in the HIV negative group. These findings were, however, not statistically significant. Propofol was received by males only and at a median dose of 30.3 mg/kg and contrary to literature, propofol requirements were also higher in patients on ARVs by 27.5 mg/kg. A statistically significant higher dose was documented on D3 with a p-value of 0.044.

### Primary outcome

Patients on ARVs required significantly higher doses of morphine and ketamine on the 2nd day of admission and ketamine on the 3rd day of admission. Interestingly fewer HIV negative patients received benzodiazepines, but had a trend to receiving a higher dose of midazolam. These findings were not statistically significant probably due to a much lower number of patients receiving benzodiazepines in the whole cohort.

### Secondary outcomes

A higher percentage of polytrauma patients were male in the age range 25–45, while females had a higher percentage of ARV use. This is in in keeping with data observed in the general population. Only 20.3% of total sample were HIV positive, of this only 93.2% were on ARVs.

**Table 2:** Summary data for cohort (*n* = 216)

		Median (IQR) or *n* (%)
**Age**		33 (26.0–43.0)
**Gender**	*F*	39 (18.1%)
	*M*	176 (81.9%)
**Weight (kg) (** *n* ** = 192)**		70 (64.3–83.0)
**GCS percentage of maximum**		73% (53 − 100%)
**HIV**	*Negative*	172 (79.6%)
	*Positive*	44 (20.4%)
**ART**	*No*	3 (6.8%)
	*Yes*	41 (93.2%)
**Liver disease**		40 (18.5%)
**Head injury**		127 (58.8%)

Table 3 provides detailed analgosedation administration data for the entire cohort. Of the 208 patients that received opioids, 207 (99.5%) received morphine, one (0.5%) patient receive fentanyl and one (0.5%) received sufentanil in addition to morphine. Of the 8 patients that did not receive an opioid, all of them received ketamine as the mainstay of their sedation, with none receiving infusions of midazolam or propofol, and one receiving boluses of clonazepam and haloperidol. Of the 50 patients who received benzodiazepines 40, (80%) received midazolam, 10 (20%) received diazepam, and one (2%) received clonazepam. All 20 patients who received propofol also received morphine and ketamine infusions. All patients who received propofol were male, *p* = 0.029. Significant differences were noted in sedation doses between males and females. Median total morphine dose in females was 144 mg (IQR, 72 mg-168 mg) as opposed to 192 mg (IQR, 120 mg-264 mg) in males, *p* = 0.002. For ketamine the corresponding figures were 1680 mg (960 mg-2400 mg) for females and 2400 mg (1440 mg-3360 mg) for males, *p* = 0.003. No statistically significant difference was noted with respect to benzodiazepines. These findings were also noted in the weight-adjusted doses. The morphine weight-adjusted dose was 1.8 mg/kg (IQR, 1.1 mg/kg-2.7 mg/kg) in females and 2.5 (IQR, 1.7 mg/kg-3.6 mg/kg) in males, *p* = 0.012. For ketamine the corresponding results were 24.6 mg/kg (14.1 mg/kg-38.0 mg/kg) in females and 33.9 mg/kg (19.2 mg/kg-48.4 mg/kg) in males, *p* = 0.009. No significant difference was noted in the weight-adjusted doses of midazolam.

**Table 3:** Analgosedation administration data (for entire cohort *n* = 216)

	Median (IQR) or *n* (%)
**Opioid administration**	208 (96.3%)
Morphine infusion dose D1 (mg)	72 (48.0–96.0)
Morphine infusion dose D2 (mg)	48 (48.0–96.0)
Morphine infusion dose D3 (mg)	48 (40.0–90.0)
Morphine boluses D1-D3 (mg)	9 (5.0–20.0)
Total morphine dose D1-D3 (mg)	168 (120.0–243.0)
**Ketamine administration**	177 (81.9%)
Ketamine infusion D1 (mg)	720 (480.0–1200.0)
Ketamine infusion D2 (mg)	720 (480.0–1200.0)
Ketamine infusion D3 (mg)	720 (480.0–1100.0)
Ketamine bolus (mg)	50 (30.0–80.0)
Total ketamine dose (mg)	2240 (1440.0–3320.0)
**Midazolam administration**	50 (23.1%)
Midazolam D1 (mg)	48 (24.0–72.0)
Midazolam D2 (mg)	48 (48.0–73.0)
Midazolam D3 (mg)	48 (24.0–72.0)
Midazolam bolus (mg)	8 (5.0–10.0)
Midazolam total dose (mg)	93 (24.0–205.0)
**Propofol administration**	20 (9.3%)
Propofol infusion D1 (mg)	840 (480.0–2400.0)
Propofol infusion D2 (mg)	860 (600.0–1320.0)
Propofol infusion D3 (mg)	960 (480.0–1680.0)
Propofol bolus (mg)	30 (30.0–60.0)
Propofol total dose (mg)	2160 (1080.0–3290.0)
**Haloperidol administration**	10 (4.6%)
Haloperidol dose (mg)	5 (5.0–5.0)
***Weight-based doses***	
Morphine (mg/kg) (*n* = 184)	2.3 (1.6–3.6)
Ketamine (mg/kg) (*n* = 159)	31.6 (17.4–47.2)
Midazolam (mg/kg) (*n* = 36)	1.1 (0.3–2.2)
Propofol_(mg/kg) (*n* = 19)	30.3 (10.7–51.4)
Haloperidol (mg/kg) (*n* = 10)	0.07 (0.06–0.08)

Table 4 provides comparisons between HIV negative patients and HIV positive patients on ARVs.

**Table 4:** Comparison between HIV negative patients and HIV positive patients on ARVs

	HIV negative	HIV positive + ARVs	
	Median (IQR) or *n* (%)	Median (IQR) or *n* (%)	*p*-value
Age	33 (24.0–44.0)	34 (30.0–41.0)	0.515
Male sex	147 (86.0%)	26 (63.4%)	< 0.001
Weight (kg)	70 (64.0–83.0)	73.3 (66.0–84.0)	0.583
GCS percentage of maximum	73% (47 − 100%)	82% (60 − 100%)	0.298
Liver disease	34 (19.8%)	6 (14.6%)	0.449
Head injury	104 (60.5%)	21 (51.2%)	0.280
**Morphine administration**	164 (95.3%)	40 (97.6%)	1.000
*Morphine infusion dose D1 (mg)*	72 (48.0–96.0)	72 (48.0–96.0)	0.124
*Morphine infusion dose D2 (mg)*	48 (48.0–72.0)	72 (48.0–120.0)	0.007
*Morphine infusion dose D3 (mg)*	48 (36.0–72.0)	72 (48.0–96.0)	0.264
*Morphine cumulative boluses D1-D3 (mg)*	6 (3.0–13.0)	25 (15.0–48.0)	0.002
*Total morphine dose D1-D3 (mg)*	168 (120.0–242.0)	216 (132.0–264.0)	0.257
**Ketamine administration**	138 (80.2%)	36 (87.8%)	0.260
*Ketamine infusion dose D1 (mg)*	720 (480.0–1200.0)	960 (540.0–1440.0)	0.311
*Ketamine infusion dose D2 (mg)*	720 (480.0–1200.0)	960 (480.0–1440.0)	0.043
*Ketamine infusion dose D3 (mg)*	600 (480.0–960.0)	960 (720.0–1440.0)	0.004
*Ketamine cumulative boluses D1-D3 (mg)*	50 (30.0–80.0)	40 (30.0–120.0)	0.907
*Total ketamine dose D1-D3 (mg)*	2160 (1320.0–3120.0)	2643 (1560.0–3685.0)	0.093
**Midazolam administration**	29 (16.9%)	11 (26.8%)	0.142
*Midazolam infusion dose D1 (mg)*	48 (24.0–72.0)	48 (0.0–120.0)	1.000
*Midazolam infusion dose D2 (mg)*	48 (48.0–72.0)	48 (24.0–73.0)	0.852
*Midazolam infusion dose D3 (mg)*	72 (48.0–90.0)	36 (24.0–60.0)	0.238
*Midazolam cumulative boluses D1-D3 (mg)*	6.5 (5.0–11.5)	10 (4.0–10.0)	1.000
*Total midazolam dose D1-D3 (mg)*	120 (24.0–216.0)	72 (15.0–193.0)	0.720
**Propofol administration**	16 (9.3%)	4 (9.8%)	1.000
*Propofol infusion dose D1 (mg)*	720 (480.0–1920.0)	2400 (240.0–2400.0)	0.800
*Propofol infusion dose D2 (mg)*	860 (480.0–1200.0)	1560 (720.0–3600.0)	0.262
*Propofol infusion dose D3 (mg)*	600 (480.0–1320.0)	3500 (2200.0–4800.0)	0.044
*Propofol cumulative boluses D1-D3 (mg)*	45 (30.0–60.0)	30 (30.0–30.0)	0.667
*Total propofol dose D1-D3 (mg)*	2160 (1260.0–3170.0)	3995 (840.0–9515.0)	0.617
**Haloperidol administration**	9 (5.2%)	1 (2.4%)	0.691
*Total haloperidol dose (mg)*	5 (5.0–5.0)	5 (5.0–5.0)	1.000
**Weight-based doses**			
*Morphine (mg/kg) (* *n* ** = 184)**	2.3 (1.6–3.4)	3.0 (1.6–3.6)	0.438
*Ketamine (mg/kg) (* *n* ** = 159)**	29.8 (17.3–45.1)	34.3 (24.7–49.3)	0.374
*Midazolam (mg/kg) (* *n* ** = 36)**	1.3 (0.4–2.2)	1.1 (0.2–2.2)	1.000
*Propofol_(mg/kg) (* *n* ** = 19)**	30.3 (17.5–42.7)	57.8 (10.1–163.6)	0.596
*Haloperidol (mg/kg) (* *n* ** = 10)**	0.07 (0.06–0.08)	0.05 (0.05–0.05)	0.200

## Discussion

There are a large number of South Africans on ARVs and thriving. The majority are young adults around the age of 33–34 years who also form the largest portion of the population of trauma victims. It is important to note that at the time of the study there was still a large number of patients who were not tested for HIV prior to admission and thus not on ARVs even though some tested positive during the admissions. This is in keeping with the current statistics at the time that SA had not reached its goal of having 90% of HIV positive people on HAART [13]. The commonest ARV regimens used included were a fixed drug combination of Tenofovir/ Emitricitabine and Efavirenz, TDF/3TC/EFV, Abacavir/Lamivudine and Dolutegravir etc.

Severe trauma often leads to ICU admission where multiple different drug therapies are necessary for Improved outcomes. Polypharmacy is of great concern in any medical discipline as it predisposes patients to negative impacts of drug to drug interactions. A national study done in Cape Town highlighted the knowledge gaps in majority of medical doctors regarding ARVs [8]. This was an online survey targeting health care workers, mainly nurses, doctors and pharmacists, who had used the HIV/AIDS and TB hotline. There were 1950 responses, of which 35.8% were doctors. Despite this study being done merely a year after DTG was named the first-line ARV agent and health care worker training done in that year, major knowledge gaps were identified specifically regarding drug interactions and consequent necessary dose adjustments [8]. This was also noted in our study as sedation practises did not have specific protocols for patients on ARVs.

Some drug interactions are not directly linked to sedation challenges in ICU; however, it leaves a question of the possibility of treatment failure of antiretrovirals and other coadministered medications. Research seeking to answer this specific question on each of the drugs will be of great value in holistic patient care and prevention of treatment failure and the devastating consequences thereof.

The main practise in TICU was using first-line analgosedatives namely morphine 1 mg/ml and ketamine 10 mg/ml at a rate of 3-5 ml/hr, titrated to effect. Midazolam and or propofol infusion was only added as 3rd line, and this study revealed that the third line agents were only received by male patients. The patients included in the study were all sedated for minimum of 72 h with controlled mechanical ventilation tailored to meet individual metabolic needs or neuroprotective targets. Due to admission criteria to this unit about 97.7% of these patients had emergency surgery mainly laparotomies (42.2%) and craniotomies (50.7%), orthopaedic procedures (44.5%), while the rest of the patients had either vascular or urology or soft tissues injuries that required debridement and suturing. The majority of these injuries were sustained due to high velocity crashes and as such most patients had multiple injuries. The usage of blood and blood products was also high due to the multiple injuries and multiple surgical procedures per patient, another contributing factor was the delays associated with moving patients from the incident scenes, to the base hospitals and finally transferred to this quaternary unit.

The study demonstrated an inconsistency in dose requirements of morphine between male and females, between patients on ARVs versus those not on ARVs and between different days during the first 72 h of admission. There was no difference between the groups on comparing the first 24 h, this could be explained by the need for heavy sedation on the first day as that is the day most patients undergo surgical procedures.

The largest portion of our study population was male, it was also noted that males whether on ARVs or not required higher doses of each of the sedatives. This phenomenon may be explained by an increased risk of alcohol in males which predisposes them to trauma and abnormal drug metabolism [14].

Patients on ARVs appeared to require slightly lower doses of midazolam; 1.1 mg/kg versus 1.3 mg/kg, however no reaching statistical significance, which was in keeping with a study done on the safety of using midazolam in patients on protease inhibitors. This was a retrospective study looking at procedural sedation for patients undergoing bronchoscopy. Both groups (patients on ARVs and those not on ARVs) were given the same dosage of midazolam and the duration it took for study participants to completely recover after the bronchoscopy was documented. That study found that coadministration of midazolam in patients on PIs resulted in severe prolonged duration of action and a subsequent 3 day longer duration of hospital stay [15].

The actual doses of propofol were significantly higher in patients on ARVs, it is likely that this did not reach statistical significance because of the small number of patients that received the propofol. This finding does not correlate with the expected drug to drug interaction as per theoretical literature published. Some authors estimated no drug interactions, while others expected that ARVs will decrease required propofol doses. [7, 16, 17]. To date there are no published studies that investigated this particular aspect of ARVs and sedatives drug-drug interactions. Previous studies on HIV trauma patients have shown no difference in ICU outcome when ARVs were not yet available in the context of trauma [18].

### Limitations

Human errors in documentation or data collection and capturing are a risk, however the study was carried out in a closed unit with very strict monitoring and titration of sedation, most clinicians and nurses were using familiar sedation guidelines/habits. Electronic data was reliable and easy to compare physician documentation and nurses reports to minimise errors. The retrospective chart analysis nature of the study and the single centre nature may minimise generalisation of findings, however similar severe trauma patients on ARVs are treated country-wide. Due to the polypharmacy, other drugs could have the same effect as ARVs on the sedatives, so confounding the results. The first day of trauma ICU care usually has surgical procedures and thus high pain scores and exposure to the anaesthetic drugs, hypovolaemic shock and other drugs given enroute to the unit. Other patients were admitted as referrals from other units in the hospital where sedation was already used.

## Conclusion and recommendations

The findings suggest that there should be dose adjustments when prescribing sedatives for patients on ARVs. A dose reduction for midazolam and slightly higher doses for morphine, ketamine and propofol may be reasonable. Further investigation of these differences would assist in optimising sedation in the critical hours of ICU care and also prevention of ARV treatment failure that is a possibility in view of these altered pharmacokinetics. A randomised clinical trial is suggested to properly assess these interactions with a larger study cohort.

## Data Availability

No datasets were generated or analysed during the current study.
